# Nursing research priorities in critical care in Brazil: Delphi Study*

**DOI:** 10.1590/1518-8345.4055.3370

**Published:** 2020-09-30

**Authors:** Adriano da Silva Acosta, Sayonara de Fátima Faria Barbosa, Grace Teresinha Marcon Dal Sasso

**Affiliations:** 1 Universidade Federal de Santa Catarina, Florianópolis, SC, Brazil.

**Keywords:** Research, Critical Care, Nursing, Delphi Tecnique, Consensus, Intensive Care Units, Pesquisa, Cuidados Críticos, Enfermagem, Técnica Delfos, Consenso, Unidade de Terapia Intensiva, Investigación, Cuidados Críticos, Enfermería, Técnica Delfos, Consenso, Unidades de Cuidados Intensivos

## Abstract

**Objective:**

to analyze the nursing research priorities in critical care in Brazil identified by specialists and researchers in the area, as well as to establish the consensus of the topics suggested by the experts.

**Method:**

a descriptive study, using the e-Delphi technique in three rounds. The research participants were 116 Brazilian nurses who are experts in critical care in the first round, ending up with 68 participants in the third round of the study. Descriptive statistics were used to analyze the demographic variables and the results of the research topics in the second round. In the final analysis, the *Kappa* agreement coefficient was calculated, comparing the answers between rounds two and three.

**Results:**

63 research topics were generated, grouped into 14 domains of intensive care practice in the first round, and consensus was settled in the subsequent rounds. Topics such as humanization of care (0.56), bloodstream infection control (0.54), and nursing care for polytrauma patients (0.51) were items rated above 0.50 in the agreement analysis between the topics in the two rounds using the *Kappa* coefficient.

**Conclusion:**

this study provides an important guideline for nursing research in critical care in Brazil, guiding for future research efforts in the area.

## Abstract (pt)

**Objetivo:**

analisar as prioridades de pesquisa em enfermagem em cuidados críticos no Brasil identificadas por especialistas e pesquisadores na área, bem como estabelecer o consenso dos tópicos sugeridos pelos *experts*.

**Método:**

estudo descritivo, utilizando a técnica e-Delphi em três rodadas. Os participantes da pesquisa foram 116 enfermeiros brasileiros *experts* em cuidados críticos na primeira rodada, finalizando com 68 participantes na terceira rodada do estudo. Estatísticas descritivas foram utilizadas para analisar as variáveis demográficas e os resultados dos tópicos de pesquisa na segunda rodada. Na análise final, foi calculado o coeficiente *Kappa* de concordância, comparando as respostas entre as rodadas dois e três.

**Resultados:**

foram gerados 63 tópicos de pesquisa agrupados em 14 domínios da prática em terapia intensiva na primeira rodada, sendo estabelecido o consenso nas rodadas subsequentes. Tópicos como humanização do cuidado (0,56), controle de infecção em corrente sanguínea (0,54) e assistência de enfermagem ao paciente politraumatizado (0,51) foram os itens com classificação acima de 0,50 na análise de concordância entre os tópicos nas duas rodadas utilizando o coeficiente *Kappa*.

**Conclusão:**

este estudo fornece uma importante diretriz para a pesquisa em enfermagem em cuidados críticos no Brasil, orientando para futuros esforços de pesquisa na área.

## Abstract (es)

**Objetivo:**

analizar las prioridades de investigación en enfermería en cuidados críticos en Brasil identificadas por especialistas e investigadores en el área, así como establecer un consenso sobre los tópicos sugeridos por expertos.

**Método:**

estudio descriptivo, con utilización de la técnica e-Delphi en tres rondas. Participaron en la investigación 116 enfermeros brasileños, expertos en cuidados críticos en la primera ronda, siendo que la tercera ronda del estudio terminó con 68 participantes. Se utilizaron estadísticas descriptivas para analizar las variables demográficas y los resultados de los tópicos de investigación en la segunda ronda. En el análisis final, se calculó el coeficiente *Kappa* de concordancia, comparando las respuestas entre las rondas dos y tres.

**Resultados:**

se generaron 63 tópicos de investigación, agrupados en 14 dominios de prácticas de cuidados intensivos en la primera ronda, y se estableció un consenso en las rondas posteriores. Tópicos como la humanización de la atención (0,56), el control de infecciones del torrente sanguíneo (0,54) y la atención de enfermería para pacientes politraumatizados (0,51) fueron los ítems clasificados por encima de 0,50 en el análisis de concordancia entre los tópicos en ambas rondas utilizando el coeficiente *Kappa*

**Conclusión:**

este estudio proporciona una importante directriz para la investigación de enfermería en cuidados críticos en Brasil, orientando los futuros esfuerzos de investigación en el área.

## Introduction

The need to involve as many people as possible in the identification and prioritization of research topics is highlighted and has been recognized by researchers. This strategy can not only guarantee that the interests of relevant knowledge groups are considered, but also meets the increase in research properties, being real that the probability of these results influence the development of the clinical practice^(^ 1 ^)^.

To achieve the greatest impact at the end of these studies, it is essential to identify priorities within intensive care research. Even with the continued development of international research, many unanswered questions remain about the prevention, diagnosis, and treatment of serious illnesses, as well as the care of critically ill patients. It is observed that research agendas have been largely determined by researchers and medical scientists, but there is a growing expectation that multidisciplinary teams will be involved in identifying clinical research priorities^(^ 2 ^)^.

Nurses constitute the largest health workforce and play key roles in improving results in the area. One of these roles is to carry out research that can support the improvement of these results, strengthening their position as protagonists that influence the health system and the generation of evidence. However, nursing research presents challenges to be overcome, which cover the category in general^(^ 3 ^)^. It is known that the specialty of critical care as an area of assistance, given its complexity and the advances, require increasingly sustained knowledge bases, highlighting the need for this assistance to be based on the results presented by research studies on the theme^(^ 4 ^)^.

Over the past 30 years, international studies on critical care research priorities have been developed, with emphasis on the studies developed in the United States, Australia, Ireland, Finland, the United Kingdom, and Hong Kong. Such analyses submitted as results the most varied research questions, due to the different cultural ideologies, associated with the influence of political and economic resources of each country. Another evidence observed is that all of these studies used some form of expert consensus method to generate priorities^(^ 5 ^)^.

Although there are review studies that present nursing research priorities in the health systems and services, no research study focusing on nursing research priorities in critical care in Brazil has been identified in the search for health journals and databases. Thus, this study was proposed with the aim of analyzing the nursing research priorities in critical care in Brazil identified by specialists and researchers in the area, as well as to establish the consensus on the topics suggested by the experts.

## Method

A descriptive and exploratory research with a quantitative nature. For the development of this study, the on-line Delphi technique was used, which is characterized by the possibility of generating consensus on a topic and occurs through a systematic communication structure, controlled by the researcher, allowing that, at the end of the rounds, consensus be reached for the problem in question^(^ 6 ^-^ 7 ^)^.

The research participants were Brazilian nurses who are specialists and researchers in critical care, being PhDs and Masters in nursing and specialists in the care practice. The sample was intentional and non-probabilistic, and the selection was made through a search on the Lattes Platform of the National Council for Scientific and Technological Development (*Conselho Nacional de Desenvolvimento Científico e Tecnológico* - CNPq), using the following keywords: “critical care”, “intensive care” and “intensive therapy”.

Regarding the selection of the participants, the relevant level of professional qualification on the thematic area to be studied was considered of extreme importance to obtain a consensus of ideas. To this end, filters were applied to the database in this search, regarding academic training, professional performance, specialties, and updating of curricula. After selecting the experts*,* the summary of all curricula found was read to confirm the performance in the theme; the existence was also verified of developed research projects or under development related to critical care, to the publication of articles in this area in the last five years, and to the performance in the area of the specialists also from at least 5 years.

For selection criterion, the professionals that had at least two of the items mentioned above were included. To ensure data representativeness, the participants were selected from all the Brazilian states. Nurses with doctoral and master degrees in areas unrelated to the topic and specialists who were not working in the area were excluded.

With the application of the participant selection procedure, a list of 422 professionals was obtained. It was decided to send the invitation to all these professionals by email by contacting the Lattes Platform, of which 116 showed interest in participating in the research, through confirmation by the Google Forms^®^ platform, validating the Free and Informed Consent Form (FICF) and answering to the first round of the study. In the 1^st^ stage of the research, an e-mail was sent with an online semi-structured questionnaire being comprised in two sections: the first sought sociodemographic data (age, gender, state of residence, length of training, professional experience, academic degree, and professional area). The second section consisted of three open questions that questioned what the research priorities were for the patients, their families and the needs of the professionals.

The answers to the questionnaire were automatically entered via the platform Google Forms^®^ to an Excel spreadsheet and later exported to the Statistical Package for Social Sciences (SPSS^®^) program for Windows, version 20.0. The sociodemographic variables were described by frequencies, means, and percentages. For the variables of dimension of research priorities aimed at the patients, their family, and the professional needs, content analysis was adopted^(^ 8 ^)^. The answers of the initial consultation process regarding the research priorities were categorized and grouped, using pre-defined keywords derived from the main research categories in the critical care literature. This process generated 63 research topics grouped into 14 domains of intensive care practice, giving rise to a new instrument for analyzing participants in the following rounds of the study.

In the 2^nd^ round, the experts were sent a new invitation with information on the continuation of the consensus process. Via this e-mail, the participant received the link for online access to the questionnaire containing the topics of research priorities that were listed by the participants in the first round of the study.

At this stage, the participants were asked to indicate their degree of agreement or disagreement with the research questions using a five point *Likert* scale (0: totally disagree, 1: partially disagree 2: indifferent, 3: partially agree, 4: totally agree), for each research priority of the instrument. The answers provided by the experts were compiled statistically, generating new feedback*,* and the criteria adopted to determine the level of consensus were based on the degree of agreement [summing up the percentage of 3 (partially agree) and 4 (totally agree) answers obtained in this round].

To establish the consensus degree of the participants to the research topics suggested by the experts, the literature indicates that establishing such consensus degree should be done by the researchers, with no rules for such^(^ 9 ^)^. In order to determine the degree of consensus of the participants, the most used statistics includes measures of central tendency, such as the median and measures of dispersion like the Interquartile Range^(^ 10 ^)^.

Therefore, a descriptive statistical treatment (relative frequency, median, and interquartile range) was chosen as a resource for the criteria to determine the degree of consensus, based on the degree of agreement [sum of the percentage of answer options 3 (partially agree) and 4 (totally agree)].

In the third (final) round, an e-mail invitation was again sent to the specialists with information on the continuation of the consensus process. In this email we send the link of the online questionnaire containing the topics of research priorities that were listed by the participants in the first round of the study, plus the level of consensus on the degree of agreement [sum of the percentage of 3 (partially agree) and 4 (totally agree) answers obtained by the responses tabulated statistically in the 2^nd^ round of the study].

In the final analysis of the third round, the statements were classified in importance by calculating the means and standard deviation. The *Kappa* agreement coefficient was calculated for all the research questions, comparing the answers of the participants between rounds two and three. For comparison purposes, the values of *Kappa* were adopted, where the strength of the agreement varies from poor to almost perfect. In summary, when the value of *Kappa* was close to 0, this meant a low agreement between the evaluators, whereas values close to 1 meant an almost perfect agreement^(^ 11 ^)^.

For interpreting the *Kappa* coefficient (standardized mean difference), the values are interpreted as following: 0 (no agreement), 0-0.19 (poor agreement), 0.20-0.39 (weak agreement), 0.40-0.59 (moderate agreement), 0.60-0.79 (substantial agreement), and greater than or equal to 0.80 (almost total agreement). The level of significance was set at <0.05^(^ 11 ^-^ 12 ^)^. The Google Forms^®^ version was selected to administer the e-Delphi questionnaires, and data analysis was performed using the Microsoft Excel software, version 16.10, and the SPSS^®^ statistical program for Windows, version 20.0.

The three-round Delphi method used in this study was collected from May to September 2018, as shown in Figure 1.

**Figure 2:**
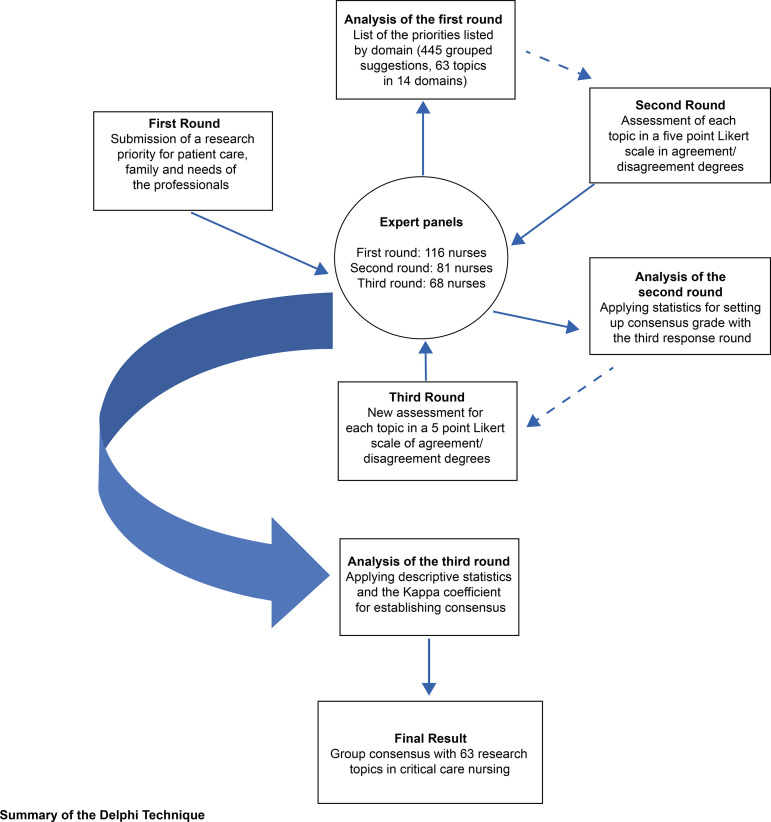
Domains of the intensive care practice based on the participants’ research suggestions. Brazil 2018

**Figure 2:** Domains of the intensive care practice based on the participants' research suggestions. Brazil 2018

Domain 1 - Related to the family	Research studies that explore the perceptions and experiences of the families of critically ill patients admitted to the ICU.
Domain 2- Related to the intensive care unit	Research studies related to the use of indicators and technologies to assist in the care of critical patients.
Domain 3 - Related to the patient's well-being in the ICU	Research on the interventions that nurses can carry out to promote the health and well-being of the patients.
Domain 4 - Related to Mechanical Ventilation (MV)	Research regarding the care provided to the patients in the prevention of injuries related to Mechanical Ventilation.
Domain 5 - Related to Sepsis/HCAI prevention	Research on the roles of nursing in controlling and preventing Health Care-Associated Infections (HCAIs) to reduce morbidity in the patients.
Domain 6 Research - Related to Hemodynamics	Research related to the activities and performance of the nurses, in relation to critical patients in the hemodynamic monitoring of the patients.
Domain 7 - Related to education	Research on the development of ICU care protocols and evidence-based practices.
Domain 8 - Related to the workforce	Research focusing on staff dimensioning and impact on the outcome of patient care in relation to the ICU workload.
Domain 9 - Related to patient safety	Research on how the safety culture and effective communication can improve care.
Domain 10 - Related to neurointensivism	Research studies regarding the role of the nurses in the face of neurocritical patients.
Domain 11 - Related to care management	Research on a variety of issues, such as the work process, management, and systematization of the nursing care for critically ill patients.
Domain 12 - Related to nursing care	Research studies related to improvements in nursing care, including interventions that would be effective in obtaining results for the patients admitted to the ICU.
Domain 13 - Related to the discharge from the ICU	Research on the involvement of relatives in palliative care and the de-hospitalization process.
Domain 14 - Related to Ethics	Research on the impact of care at the end of life of the patients and decision making by the nursing team.

The ethical recommendations were followed and the research was approved by the Research Ethics Committee, through the Certificate of Presentation for Ethical Appreciation (*Certificado de Apresentação para Apreciação* Ética, CAAE) No. 80734317.5.0000.0121. The FICF was submitted online to the participants before starting data collection, through a clarification page about the research. The participants needed to click on the “I agree to participate in the survey” option to confirm their agreement with the terms of the study and be directed to the next screen with the questionnaire.

## Results

One hundred and sixteen nurses with expertise in critical care answered the questionnaire of the first round. In the second round 81/116 (69%) participants responded and, in the third round, 68/81 (84%). With regard to the sociodemographic data, 75.8% of the participants were female and 53% of the sample had a Master’s degree as their highest degree. The age of the participants ranged from 27 to 60 years old with a mean of 41.9. As for graduation time, there was a fluctuation between 5 and 40 years, with a mean of 18 years among the participants. Regarding the time of experience in critical care, the participants reported 3 to 35 years, with predominance in the range between 6 to 15 years (47.63%). The main area of activity cited among the participants was teaching (52.5%). Among the workplaces of the participants, there is an emphasis on educational institutions (59.3%) and public health institutions (36.3%). As for the regions where the participants worked, there was a predominance of the Southeast (38.8%) and South (28.5%) regions, due to the greater presence of health and educational institutions in these areas in the national territory. The sociodemographic characteristics of the study participants in the three rounds are shown in the Table 1.

**Table 1:** Characterization of research participants regarding sociodemographic aspects. Brazil, 2018

Variable	1st round	2nd round	3rd round
	n=116	n=81	n=68
Age; n (%)			
≤ 30 years old	8 (6.9)	7 (8.7)	6 (8.8)
31 to 40 years old	46 (39.7)	30 (37.0)	24 (35.3)
41 to 50 years old	39 (33.6)	29 (35.8)	23 (33.8)
51 to 60 years old	23 (19.8)	15 (18.5)	15 (22.1)
Time of graduation training; n (%)			
≤ 10 years	24 (20.7)	18 (22.2)	15 (22.1)
11 to 20 years	47 (40.5)	30 (37.0)	25 (36.7)
21 to 30 years	32 (27.6)	25 (30.9)	20 (29.5)
31 to 40 years	13 (11.2)	8 (9.9)	8 (11.8)
Time of experience in critical care; n (%)			
≤ 5 years	8 (6.9)	8 (9.9)	7 (10.3)
6 to 15 years	62 (53.4)	38 (46.9)	29 (42.6)
16 to 25 years	35 (30.2)	27 (33.3)	24 (35.3)
26 to 35 years	11 (9.5)	8 (9.9)	8 (11.8)
Gender			
Female; n (%)	89 (76.7)	59 (72.8)	53 (77.9)
Male; n (%)	27 (23.3)	22 (27.2)	15 (22.1)
Degree			
Post-Doctorate; n (%)	8 (6.9)	5 (6.2)	4 (5.9)
Doctorate; n (%)	36 (31.1)	25 (30.9)	19 (27.9)
Master's Degree; n (%)	57 (49.1)	44 (54.3)	39 (57.4)
Specialization, n (%)	15 (12.9)	7 (8.6)	6 (8.8)
Main workplace			
Private Education Institution; n (%)	34 (29.3)	25 (30.9)	18 (26.5)
Public Education Institution; n (%)	39 (33.6)	24 (29.6)	19 (27.9)
Private Health Institution; n (%)	6 (5.2)	3 (3.7)	3 (4.4)
Public Health Institution; n (%)	37 (31.9)	29 (35.8)	28 (41.2)
Main work activity			
Assistance; n (%)	46 (39.7)	34 (42.0)	33 (48.5)
Teaching; n (%)	65 (56.0)	44 (54.3)	32 (47.1)
Research; n (%)	5 (4.3)	3 (3.7)	3 (4.4)
Region of professional activity			
Midwest; n (%)	8 (6.9)	5 (6.2)	3 (4.4)
Northeast; n (%)	26 (22.4)	16 (19.8)	15 (22.1)
North; n (%)	8 (6.9)	4 (4.9)	3 (4.4)
Southeast; n (%)	40 (34.5)	33 (40.7)	28 (41.2)
South; n (%)	34 (29.3)	23 (28.4)	19 (27.9)

In the first round, 445 research topics were suggested aimed at the patients, their families and the needs of the professionals in the field. The suggestions were organized and grouped into major domains. For example, the effect of the extended visit in the Intensive Care Unit (ICU), the communication of difficult news, and the situational clarification of the treatment were grouped in the domain related to the family. Using this content analysis process, the list of 445 suggestions was reduced to 63 research topics grouped into 14 domains of intensive care practice. From the research topics identified, the following definitions were created for each domain of intensive care practice as shown in Figure 2.

Among the domains displayed in the first round of the study, there is an emphasis on the one related to the family with 12.80% (n=57), with the topic of “Reception and support to the relative in the ICU” being the most mentioned. Another topic well listed by the participants was in relation to patient safety in the ICU, with this domain accounting for 10.33% (n=46) of the research questions indicated by the participants in the first round. Topics such as “Humanization of care in the ICU” and “The role and involvement of the family in palliative care at discharge” were also well cited by the participants in the first round of the study.

In the second round of the study, of the 63 topics that were grouped into 14 domains of the intensive care practice suggested by the participants in the first round of the research, 41 (65%) reached a very high consensus, as they presented an agreement greater than 80%, a median of 4, and interquartile range of 0, as shown in Table 2.

**Table 2:** Distribution of the research topics by very high consensus agreement in the 2^nd^Delphi round. Brazil, 2018

D*	Research topics	%^†^	Md^‡^	IQR^§^
1	Approach to the brain death (BD) patient's family	93.8	4	0
1	Communication of bad news	95.1	4	0
1	Effect of extended visit in the ICU	95.1	4	0
2	ICU severity indicators	96.3	4	0
2	Care technologies in a critical environment	97.5	4	0
2	Predictors of mortality in the ICU	93.8	4	0
3	ICU pain assessment and management scales	96.3	4	0
3	Comfort conditions for patients in the ICU	97.5	4	0
3	Nursing prevention/interventions in relation to Pressure Injury	91.4	4	0
4	Prevention interventions for Ventilation-Associated Pneumonia	97.6	4	0
4	Oral care for ICU intubated patients	96.3	4	0
4	Nursing interventions for patients on MV||	97.6	4	0
5	Interventions to reduce HCAI^¶^ in the ICU	98.8	4	0
5	Control/Prevention of bloodstream infection	98.8	4	0
6	Cardio Pulmonary Resuscitation (CPR)	97.5	4	0
6	Nursing interventions in invasive monitoring	96.3	4	0
6	Care in the administration of Vasoactive Drugs	95.1	4	0
7	Development of preventive care protocols	97.5	4	0
7	Evidence-based practices in intensive care	97.5	4	0
8	Staff dimensioning in the ICU	98.8	4	0
8	Workload and its impact on the outcome of care	98.8	4	0
9	ICU patient safety	97.5	4	0
9	Safety culture in the ICU	97.5	4	0
9	Safety in the administration of high risk medications	97.5	4	0
9	Effective ICU communication	98.8	4	0
9	Biosafety in the ICU	95.1	4	0
10	Neurocritical patient care	96.3	4	0
10	Neurological assessment in the ICU	97.5	4	0
10	Organ donation/transplants	93.8	4	0
10	Maintenance of potential organ and tissue donors	93.8	4	0
11	ICU work process	96.3	4	0
11	High performance ICU management	97.5	4	0
11	Systematization of the Nursing Care	93.8	4	0
11	Critical patient-centered nursing care	95.1	4	0
12	Nursing care for cardiac patients in the ICU	96.3	4	0
12	Nursing care for the patient with renal complications	97.5	4	0
12	Quality assessment of critical patient care	96.3	4	0
12	Nursing care for polytrauma patients in the ICU	96.3	4	0
13	The role of the family in palliative care at discharge	95.1	4	0
14	Ethical decision-making in the nursing practice	96.3	4	0
14	Process of death and dying/terminality in the ICU	95.1	4	0

* D = Domain of intensive care practice,

† % = Agreement on the research topics;

‡ Md = Median,

§ IQR = Interquartile range;

|| MV = Mechanical ventilation;

¶ HCAI = Health care-associated infections

Therefore, the choice was a descriptive statistical treatment, using the criteria to determine the degree of consensus, based on the degree of agreement [sum of the percentage of answer options 3 (I partially agree) and 4 (I totally agree)], in the Median and in the interquartile range.

For this, a very high consensus was considered for topics that obtained an agreement equal to or greater than 80%, a median of 4, and Interquartile Interval of 0. For the high consensus, we considered an agreement greater than 80%, a median equal to or greater than 3, and Interquartile Interval of 1.

After ending the 3^rd^ round of the study, the means and standard deviations were calculated for each research topic in the two rounds, with 12 topics classified with a mean >3.80 and with a standard deviation ranging from 0.29 to 0.7. Humanization of care in the ICU (0.56), bloodstream infection control (0.54), and nursing care for polytrauma patients (0.51) were the items rated above 0.50 in the agreement analysis between the topics in the two rounds, using the *Kappa* coefficient, being that nine topics obtained a moderate agreement classification between the rounds of consensus according to Table 3.

**Table 3:** Distribution of the research topics in domains with moderate agreement, according to the *Kappa* coefficient, based on the 2^nd^ and 3^rd^rounds. Brazil, 2018

Domains and research topics	2nd round	3rd Round	Kappa^†^	p^‡^
	Mean±SD*	Mean±SD*		
Domain 3 - Related to the patient's well-being				
Comfort conditions for patients in the ICU	3.88+0.53	3.91+0.33	0.47	0.001
Humanization of care in the ICU	3.62+0.85	3.68+0.7	0.54	0.001
Domain 4 - Related to Ventilation				
Nursing interventions for the MV patient	3.85+0.55	3.87+0.38	0.41	0.001
Domain 5 - Related to Sepsis/Prevention of HCAI^§^				
Control/Prevention of bloodstream infection	3.85+0.55	3.87+0.38	0.56	0.001
Permanence of invasive devices in the ICU	3.63+0.69	3.69+0.6	0.44	0.001
Domain 6 - Related to Hemodynamics				
Therapeutic hypothermia after cardiac arrest	3.5+0.74	3.6+0.63	0.41	0.001
Domain 11 - Related to care management				
Systematization of the Nursing Care	3.66+0.73	3.66+0.64	0.41	0.001
Domain 12 - Related to the nursing care				
Nursing care for the older adult patient in the ICU	3.66+0.68	3.68+0.58	0.43	0.001
Nursing care for polytrauma patients in the ICU	3.76+0.63	3.76+0.46	0.51	0.001

* SD = Standard deviation;

† Kappa = Kappa coefficient;

‡ p = p-value significance;

§ HCAI = Health care-associated infections

## Discussion

This is the first study to identify nursing research priorities in critical care in Brazil. Nurses with expertise in critical care prioritized fundamental issues of nursing care for critically ill patients and in supporting their families, in the context of hospitalization in critical care units. The organizational and professional issues related to the unit were also identified as priority research areas. It is worth highlighting that these priorities are similar to the research priorities previously identified in other studies carried out by several critical care organizations, with prominence in the world scenario, referring to the theme^(^ 2 ^,^ 6 ^,^ 13 ^-^ 14 ^)^.

Another important aspect to be emphasized is that all the studies developed about the research priorities in nursing in critical care used the Delphi technique to establish consensus among specialists to identify and generate research priorities^(^ 2 ^,^ 6 ^,^ 13 ^-^ 15 ^)^.

The main nursing research priorities identified in this study refer to the development of care protocols in the ICU, to the workload and its impact on the outcome of care, on the care technologies in a critical environment, on the assessment scales and on pain management, on the conditions of comfort for the patient, on the interventions to reduce HCAI, and on the control of bloodstream infections, as well as topics related to patient safety, with a focus on effective communication and administration of high surveillance drugs.

It is not surprising that topics related to patient safety have been ranked among the critical nursing research priorities in this study. Patient safety is a global issue that involves concerns related to critical incidents, such as adverse events and health care-related infections^(^ 16 ^)^. Therefore, it is crucial to support research activities aimed at developing effective programs to improve patient safety practices^(^ 17 ^)^. Adverse Events (AEs), that is, harms caused to the patient during health care, are among the top five causes of death in the United States of America and Brazil, of which their majority were preventable. From this assessment, such harms must not be exempt from a scientific approach, as the recognition of the AEs linked to the death of patients can increase the awareness of the professionals and investments in research and prevention on the theme^(^ 18 ^-^ 19 ^)^.

Among the several studies published with regard to patient safety, emphasis is placed on the approach to assessing the culture of patient safety. These assessments make up the basis for identifying areas for improvement and interventions to be carried out. Therefore, it is essential that these instruments demonstrate acceptable levels of reliability and validity when studied^(^ 20 ^)^. The development of these research studies presents results that, in the medium term, help guide the direction of the safety policies, easing the construction of a positive safety culture, committed to patient safety^(^ 21 ^-^ 22 ^)^.

Likewise, HCAIs offer challenges to patient safety, in particular the variation in the incidence of methicillin-resistant *Staphylococcus aureus*. Some government initiatives have been taken, such as the National HCAI Prevention and Control Program. In this sense, in order to improve the monitoring of the HCAIs and present national data, bulletins entitled “Patient Safety and Quality in Health Services” are being published, focusing on data related to primary bloodstream infection associated with the use of the catheter central venous and surgical site infections^(^ 23 ^)^.

Related to the topics that covered patient safety, there is another domain well evaluated by the participants that presented the use of preventive care protocols. The protocols aim to reduce variation and improve the efficiency of the practices, minimizing the influence of the subjectivity of judgment and experience, seeking to apply objectivity in care^(^ 24 ^)^. The adoption of these protocols generates standardized care and in accordance with technical-scientific parameters instituted and accepted by the scientific community^(^ 25 ^)^. In the ICU, it is of outmost importance that the nursing team, which is responsible for most of the procedures, knows and understands measures to prevent infections and specifically Ventilation-Associated Pneumonia (VAP). The risk for VAP is associated with several variables such as: malnutrition, dental diseases, traumatic injuries, immuno-suppression, and previous exposure to antibiotic therapy. The use of bundles for care/prevention can be mentioned, which have measures that, when put into practice together, allow for a great chance of decreasing VAP acquisition^(^ 26 ^-^ 27 ^)^. In a recent study, the association of a learning strategy with a bundle of care for critically ill patients undergoing mechanical ventilation showed a decrease in the incidence rate of sustained VAP over the time of the experience^(^ 28 ^)^.

Regarding the conditions of comfort to the patient in the ICU, although they are current themes and constantly discussed in the scientific literature, the measures of comfort and communication, translated into the process of humanization of care, continue as an ideal discourse, but very distant from the reality of the users and health workers. Although comfort is fundamental to the patient’s experience, the concept of comfort is still poorly defined by the professionals who provide the care^(^ 29 ^)^. A number of studies on the theme reveal that the most implemented comfort measures aim at relieving strategies for the comfort of the patients, and greater presence of relatives, as well as actions and behaviors of the team^(^ 30 ^)^.

Among the comfort-promoting strategies analyzed, those that determine general consensus in the primary studies analyzed were the management of analgesia/sedation, the performance of passive exercises, and the implementation of structured information programs, in order to provide a more humane nursing practice, which sees individuals as beings with their own experiences, even when these cannot be expressed in words^(^ 29 ^-^ 30 ^)^.

The topics related to nursing care provided to polytrauma patients and to the older adults also reached a moderate consensus in the study. A number of studies point to the use of care technologies in the nursing care practice for polytrauma patients, and the nurses’ concern about providing more targeted, effective, and immediate care is evident^(^ 31 ^)^. Intensive care units seek to achieve the best results through excellence in patient care, based on evidence, updated technology, and partnerships with teaching and research^(^ 32 ^)^. In addition, these studies highlight that the units dedicated to trauma have standardized protocols for the management of these patients, showing better results, especially in polytrauma patients with traumatic brain injury^(^ 31 ^-^ 32 ^)^.

As for the older adult patient, a qualitative study revealed that there are several obstacles to be overcome to improve care for older adult patients in the ICU, such as inadequate environments, lack of resources, and lack of knowledge and skills^(^ 33 ^)^. It is noticed that the changes related to aging associated with the worsening of clinical conditions resulting from chronic diseases have increased the incidence of hospitalizations of the older adults^(^ 34 ^)^.

Diverse review studies point out the importance of the care provided to these patients, given the susceptibility to infections, vulnerability to incidents such as falls, and increased anxiety due to the prolonged hospital stay. Another finding is a gap in the production of research studies that seek to investigate nursing care for the older adult hospitalized in the ICU, in order to contribute to the robustness of the research on the theme and to the improvement of the care practice^(^ 34 ^-^ 35 ^)^.

It is worth highlighting that the results presented are intended to develop a proposal for a national agenda of research priorities in critical care; however, as these issues are dynamic and may change over time, they need to be reviewed in the future.

Some limitations of this study need to be recognized. One of the weaknesses found that we can consider was the number of participants because, despite being a persuasive number in relation to the studies carried out by the Delphi technique, we believe that the number of nurses with expertise in critical care could have been greater, given the number of professionals selected by the Lattes Platform, of the CNPq.

Another limitation of the study was the variation in the number of nurses by region, with some states not being represented in this research. All the efforts were made to obtain a representative sample at the national level. However, this has not become feasible for all the states due to the nurses’ non-agreement to participate and stay in the research during the three rounds, and a probable absence of a *curriculum* availed on the Lattes Platform by some professionals.

The results of this study contribute to provide visibility to the themes considered priority for nursing research in critical care and thus support the development of research that improves not only the clinical practice, but also meets the needs of the professionals and the relatives. In addition, it can encourage collaborative initiatives that can be used to advance research in the area in different regions of Brazil.

## Conclusion

Delphi studies focused on establishing research priorities have become a useful way of proposing research agendas in several countries. From Brazilian nurses with expertise in the critical care area, it was possible to identify and prioritize research questions, providing a guideline on the topics of greatest interest on the part of the nurses in the national territory.

The definition of nursing research priorities in critical care is the first step to start a reflection on these topics, establishing research priorities in each related domain throughout the study.

Thus, it is considered that establishing the consensus presented in this research can contribute to minimize the academy-practice gap, allowing for research needs to be achieved according to the professional focus. Likewise, among nurse researchers, these questions can be used to define future research efforts.

In addition, it is considered that these results can contribute at the international level, given that there is a global need to establish research programs that focus on priority areas related to national health priorities.
